# 1-(2-Hy­droxy-5-methyl­phen­yl)-3-(2-methyl­phen­yl)prop-2-en-1-one

**DOI:** 10.1107/S1600536811018381

**Published:** 2011-05-25

**Authors:** D. Vijay Kumar, G. B. Thippeswamy, B. S. Jayashree, M. A. Sridhar

**Affiliations:** a Department of Pharmaceutical Chemistry, Manipal College of Pharmaceutical Sciences, Manipal 576 104, India; b Department of Studies in Physics, Manasagangotri, University of Mysore, Mysore 570 006, India

## Abstract

In the title compound, C_17_H_16_O_2_, the dihedral angle between the aromatic rings is 5.12 (13)° and an intra­molecular O—H⋯O hydrogen bond generates an *S*(6) ring.

## Related literature

For a related structure and background references to chalcones, see: Thippeswamy *et al.* (2011 ▶).
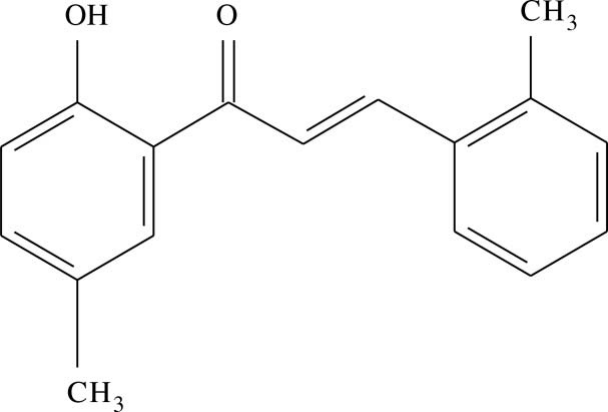


## Experimental

### 

#### Crystal data

C_17_H_16_O_2_
*M* *_r_* = 252.30
Orthorhombic, 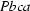

*a* = 13.3930 (11) Å
*b* = 14.1740 (16) Å
*c* = 14.5710 (15) Å
*V* = 2766.0 (5) Å^3^
*Z* = 8
Mo *K*α radiation
μ = 0.08 mm^−1^
*T* = 293 K
0.29 × 0.27 × 0.25 mm

#### Data collection

MacScience DIPLabo 32001 diffractometer
8036 measured reflections
2422 independent reflections
1654 reflections with *I* > 2σ(*I*)
*R*_int_ = 0.044

#### Refinement

*R*[*F* ^2^ > 2σ(*F* ^2^)] = 0.073
*wR*(*F* ^2^) = 0.209
*S* = 1.15
2422 reflections
175 parameters
H-atom parameters constrained
Δρ_max_ = 0.37 e Å^−3^
Δρ_min_ = −0.38 e Å^−3^

### 

Data collection: *XPRESS* (MacScience, 2002 ▶); cell refinement: *SCALEPACK* (Otwinowski & Minor, 1997 ▶); data reduction: *DENZO* (Otwinowski & Minor, 1997 ▶) and *SCALEPACK*; program(s) used to solve structure: *SHELXS97* (Sheldrick, 2008 ▶); program(s) used to refine structure: *SHELXL97* (Sheldrick, 2008 ▶); molecular graphics: *ORTEPII* (Johnson, 1976 ▶); software used to prepare material for publication: *PLATON* (Spek, 2009 ▶).

## Supplementary Material

Crystal structure: contains datablocks I, global. DOI: 10.1107/S1600536811018381/hb5868sup1.cif

Structure factors: contains datablocks I. DOI: 10.1107/S1600536811018381/hb5868Isup2.hkl

Supplementary material file. DOI: 10.1107/S1600536811018381/hb5868Isup3.cml

Additional supplementary materials:  crystallographic information; 3D view; checkCIF report

## Figures and Tables

**Table 1:** Hydrogen-bond geometry (Å, °)

*D*—H⋯*A*	*D*—H	H⋯*A*	*D*⋯*A*	*D*—H⋯*A*
O18—H18⋯O11	0.82	1.89	2.608 (3)	146

**Fig. 1.**
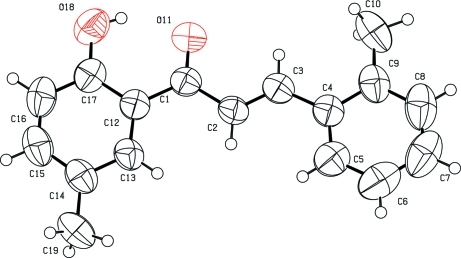
Molecular structure of (I) with 50% probability displacement ellipsoids.

**Fig. 2.**
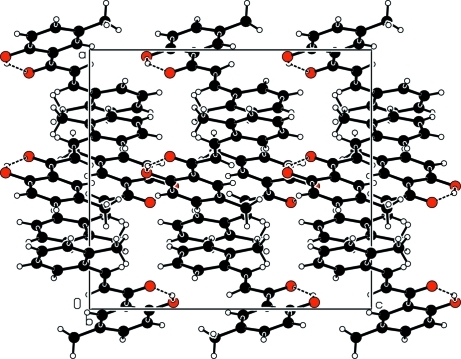
Packing diagram for (I).

## supplementary crystallographic information

### Comment

As part of our ongoing structural studies of chalcones (Thippeswamy *et al.*, 2011), we now report the synthesis and crystal structure of the title compound, (I), (Fig. 1).

The title compound, C_17_H_16_O_2_, consists of two methylphenyl rings attached at ei-ther sides of the propanone chain. The propanone unit is planar, which is confirmed by the r.m.s. deviation of 0.003 (3)Å from the mean plane. The dihedral angle between the least squares planes of 5-methylphenyl ring and 2-methylphenyl ring is 5.93 (13)° which indicates that 5-methylphenyl ring in +Syn-periplanar conformation with 2-methylphenyl ring. The bond lengths C1—O11, C1—C12, C1—C2, C2—C3, C3—C4 and bond angles C1—C2—C12,C1—C2—C3 are in good agreement with these of a similar compound reported earlier (Thippeswamy *et al.*, 2011). The angles C2—C1—O11, C12—C1—O11 and C2—C1—C12 are 119.5 (2)°, 119.4 (2)° and 121.1 (2)° respectively which indicate that the position of C1 atom is nearly in trigonal geometry. An intramolecular O—H···O hydrogen bond occurs (Table 1).

### Experimental

The title compound was prepared by dissolving 2-hydroxy-5- methoxyacetophenone 0.05 m mol in 15 ml of ethanol taken in a conical flask. To this 5 ml of 20° aqueous sodium hydroxide was added and kept for stirring at room temperature. To this mixture, 4-methylbenzaldehyde 0.05 m mol was added and continued stirring till the completion of reaction. The progress of the reaction was monitored by TLC using n-hexane and ethylacetate as solvent system. After completion of the reaction, the mixture was poured into ice cold water, mixed properly and acidified with dilute hydrochloric acid. The title compound separates as precipitate which was collected by filtration and crystallized from methanol as orange blocks of (I).

### Crystal data

**Table:**

C_17_H_16_O_2_	*F*(000) = 1072
*M**_r_* = 252.30	*D*_x_ = 1.212 Mg m^−^^3^
Orthorhombic, *P**b**c**a*	Mo *K*α radiation, λ = 0.71073 Å
Hall symbol: -P 2ac 2ab	Cell parameters from 8036 reflections
*a* = 13.3930 (11) Å	θ = 2.4–25.0°
*b* = 14.1740 (16) Å	µ = 0.08 mm^−^^1^
*c* = 14.5710 (15) Å	*T* = 293 K
*V* = 2766.0 (5) Å^3^	Block, orange
*Z* = 8	0.29 × 0.27 × 0.25 mm

### Data collection

**Table:**

MacScience DIPLabo 32001 diffractometer	1654 reflections with *I* > 2σ(*I*)
Radiation source: fine-focus sealed tube	*R*_int_ = 0.044
graphite	θ_max_ = 25.0°, θ_min_ = 2.5°
Detector resolution: 10.0 pixels mm^-1^	*h* = −15→15
ω scans	*k* = −15→16
8036 measured reflections	*l* = −17→17
2422 independent reflections	

### Refinement

**Table:**

Refinement on *F*^2^	Secondary atom site location: difference Fourier map
Least-squares matrix: full	Hydrogen site location: inferred from neighbouring sites
*R*[*F*^2^ > 2σ(*F*^2^)] = 0.073	H-atom parameters constrained
*wR*(*F*^2^) = 0.209	*w* = 1/[σ^2^(*F*_o_^2^) + (0.1131*P*)^2^ + 0.2932*P*] where *P* = (*F*_o_^2^ + 2*F*_c_^2^)/3
*S* = 1.15	(Δ/σ)_max_ = 0.009
2422 reflections	Δρ_max_ = 0.37 e Å^−^^3^
175 parameters	Δρ_min_ = −0.38 e Å^−^^3^
0 restraints	Extinction correction: *SHELXL97* (Sheldrick, 2008), Fc^*^=kFc[1+0.001xFc^2^λ^3^/sin(2θ)]^-1/4^
Primary atom site location: structure-invariant direct methods	Extinction coefficient: 0.025 (4)

### Special details

**Table:**

Geometry. All esds (except the esd in the dihedral angle between two l.s. planes) are estimated using the full covariance matrix. The cell esds are taken into account individually in the estimation of esds in distances, angles and torsion angles; correlations between esds in cell parameters are only used when they are defined by crystal symmetry. An approximate (isotropic) treatment of cell esds is used for estimating esds involving l.s. planes.
Refinement. Refinement of *F*^2^ against ALL reflections. The weighted *R*-factor *wR* and goodness of fit *S* are based on *F*^2^, conventional *R*-factors *R* are based on *F*, with *F* set to zero for negative *F*^2^. The threshold expression of *F*^2^ > σ(*F*^2^) is used only for calculating *R*-factors(gt) *etc*. and is not relevant to the choice of reflections for refinement. *R*-factors based on *F*^2^ are statistically about twice as large as those based on *F*, and *R*- factors based on ALL data will be even larger.

### Fractional atomic coordinates and isotropic or equivalent isotropic displacement parameters (Å^2^)

**Table:**

	*x*	*y*	*z*	*U*_iso_*/*U*_eq_	
C1	0.93869 (16)	0.33849 (16)	0.36305 (17)	0.0641 (7)	
C2	0.91789 (15)	0.39731 (16)	0.44755 (17)	0.0667 (7)	
H2	0.9447	0.3787	0.5036	0.080*	
C3	0.86192 (16)	0.47546 (16)	0.44390 (17)	0.0657 (7)	
H3	0.8382	0.4919	0.3861	0.079*	
C4	0.83271 (15)	0.53964 (17)	0.52070 (17)	0.0686 (7)	
C5	0.8504 (2)	0.5140 (2)	0.6137 (2)	0.0882 (9)	
H5	0.8804	0.4563	0.6266	0.106*	
C6	0.8230 (3)	0.5747 (3)	0.6874 (2)	0.1102 (11)	
H6	0.8356	0.5570	0.7477	0.132*	
C7	0.7767 (3)	0.6617 (3)	0.6682 (3)	0.1179 (14)	
H7	0.7587	0.7019	0.7159	0.141*	
C8	0.7581 (2)	0.6874 (2)	0.5779 (3)	0.1063 (12)	
H8	0.7271	0.7449	0.5665	0.128*	
C9	0.78464 (17)	0.62923 (19)	0.5019 (2)	0.0812 (8)	
C10	0.7635 (2)	0.6625 (2)	0.4032 (3)	0.1076 (11)	
H10A	0.7293	0.7220	0.4048	0.161*	
H10B	0.7225	0.6167	0.3725	0.161*	
H10C	0.8254	0.6695	0.3707	0.161*	
O11	0.91143 (13)	0.36828 (12)	0.28533 (12)	0.0830 (6)	
C12	0.98967 (15)	0.24407 (16)	0.37022 (17)	0.0626 (6)	
C13	1.02359 (15)	0.20750 (17)	0.45520 (17)	0.0673 (7)	
H13	1.0135	0.2428	0.5082	0.081*	
C14	1.07221 (17)	0.11945 (19)	0.4623 (2)	0.0772 (8)	
C15	1.0858 (2)	0.0661 (2)	0.3793 (2)	0.0908 (9)	
H15	1.1180	0.0081	0.3825	0.109*	
C16	1.0530 (2)	0.0979 (2)	0.2950 (2)	0.0924 (9)	
H16	1.0623	0.0611	0.2429	0.111*	
C17	1.00483 (19)	0.18723 (18)	0.2883 (2)	0.0749 (7)	
O18	0.97406 (16)	0.21620 (15)	0.20261 (13)	0.1000 (7)	
H18	0.9475	0.2682	0.2066	0.150*	
C19	1.1099 (2)	0.0842 (2)	0.5547 (2)	0.0995 (10)	
H19A	1.1807	0.0944	0.5588	0.149*	
H19B	1.0960	0.0181	0.5605	0.149*	
H19C	1.0770	0.1180	0.6031	0.149*	

### Atomic displacement parameters (Å^2^)

**Table:**

	*U*^11^	*U*^22^	*U*^33^	*U*^12^	*U*^13^	*U*^23^
C1	0.0505 (11)	0.0700 (14)	0.0717 (16)	−0.0065 (10)	−0.0030 (10)	−0.0002 (12)
C2	0.0521 (12)	0.0733 (14)	0.0748 (17)	0.0036 (11)	−0.0079 (11)	−0.0016 (12)
C3	0.0485 (11)	0.0733 (14)	0.0754 (16)	−0.0053 (11)	0.0020 (10)	0.0045 (12)
C4	0.0436 (11)	0.0783 (15)	0.0837 (19)	−0.0045 (11)	0.0050 (11)	−0.0046 (12)
C5	0.0648 (15)	0.109 (2)	0.091 (2)	−0.0065 (14)	0.0051 (14)	−0.0096 (17)
C6	0.0821 (19)	0.159 (3)	0.089 (2)	−0.025 (2)	0.0116 (17)	−0.026 (2)
C7	0.081 (2)	0.140 (3)	0.133 (3)	−0.025 (2)	0.036 (2)	−0.057 (3)
C8	0.0632 (16)	0.095 (2)	0.161 (4)	−0.0030 (15)	0.030 (2)	−0.030 (2)
C9	0.0486 (13)	0.0778 (16)	0.117 (2)	−0.0026 (12)	0.0137 (13)	−0.0063 (16)
C10	0.0789 (18)	0.094 (2)	0.150 (3)	0.0232 (17)	0.0071 (19)	0.0232 (19)
O11	0.0850 (12)	0.0894 (12)	0.0746 (13)	0.0041 (10)	−0.0114 (9)	0.0032 (9)
C12	0.0476 (11)	0.0683 (13)	0.0721 (16)	−0.0059 (10)	0.0035 (10)	−0.0023 (11)
C13	0.0451 (11)	0.0729 (14)	0.0839 (18)	0.0025 (11)	0.0032 (11)	−0.0028 (12)
C14	0.0506 (13)	0.0789 (16)	0.102 (2)	0.0035 (12)	0.0047 (12)	0.0072 (15)
C15	0.0686 (16)	0.0779 (17)	0.126 (3)	0.0118 (14)	0.0164 (17)	−0.0007 (17)
C16	0.0861 (18)	0.0858 (19)	0.105 (2)	0.0037 (15)	0.0221 (17)	−0.0233 (16)
C17	0.0698 (15)	0.0801 (16)	0.0749 (18)	−0.0078 (13)	0.0117 (12)	−0.0047 (13)
O18	0.1154 (16)	0.1075 (15)	0.0771 (14)	0.0001 (12)	0.0056 (11)	−0.0128 (11)
C19	0.0693 (16)	0.103 (2)	0.126 (3)	0.0179 (16)	−0.0041 (16)	0.0227 (18)

### Geometric parameters (Å, °)

**Table:**

C1—O11	1.263 (3)	C10—H10A	0.9600
C1—C12	1.506 (3)	C10—H10B	0.9600
C1—C2	1.513 (3)	C10—H10C	0.9600
C2—C3	1.339 (3)	C12—C13	1.417 (3)
C2—H2	0.9300	C12—C17	1.454 (4)
C3—C4	1.494 (3)	C13—C14	1.411 (3)
C3—H3	0.9300	C13—H13	0.9300
C4—C5	1.423 (4)	C14—C15	1.438 (4)
C4—C9	1.450 (4)	C14—C19	1.523 (4)
C5—C6	1.423 (4)	C15—C16	1.379 (4)
C5—H5	0.9300	C15—H15	0.9300
C6—C7	1.409 (5)	C16—C17	1.425 (4)
C6—H6	0.9300	C16—H16	0.9300
C7—C8	1.387 (5)	C17—O18	1.378 (3)
C7—H7	0.9300	O18—H18	0.8200
C8—C9	1.425 (4)	C19—H19A	0.9600
C8—H8	0.9300	C19—H19B	0.9600
C9—C10	1.541 (4)	C19—H19C	0.9600
			
O11—C1—C12	119.4 (2)	H10A—C10—H10B	109.5
O11—C1—C2	119.5 (2)	C9—C10—H10C	109.5
C12—C1—C2	121.1 (2)	H10A—C10—H10C	109.5
C3—C2—C1	121.8 (2)	H10B—C10—H10C	109.5
C3—C2—H2	119.1	C13—C12—C17	118.0 (2)
C1—C2—H2	119.1	C13—C12—C1	122.1 (2)
C2—C3—C4	128.4 (2)	C17—C12—C1	119.9 (2)
C2—C3—H3	115.8	C14—C13—C12	122.4 (2)
C4—C3—H3	115.8	C14—C13—H13	118.8
C5—C4—C9	118.5 (2)	C12—C13—H13	118.8
C5—C4—C3	120.9 (2)	C13—C14—C15	117.5 (3)
C9—C4—C3	120.6 (2)	C13—C14—C19	120.5 (3)
C4—C5—C6	121.4 (3)	C15—C14—C19	122.0 (2)
C4—C5—H5	119.3	C16—C15—C14	122.4 (3)
C6—C5—H5	119.3	C16—C15—H15	118.8
C7—C6—C5	119.6 (3)	C14—C15—H15	118.8
C7—C6—H6	120.2	C15—C16—C17	119.7 (3)
C5—C6—H6	120.2	C15—C16—H16	120.1
C8—C7—C6	119.7 (3)	C17—C16—H16	120.1
C8—C7—H7	120.1	O18—C17—C16	117.6 (3)
C6—C7—H7	120.1	O18—C17—C12	122.5 (2)
C7—C8—C9	122.7 (3)	C16—C17—C12	119.9 (3)
C7—C8—H8	118.6	C17—O18—H18	109.5
C9—C8—H8	118.6	C14—C19—H19A	109.5
C8—C9—C4	118.1 (3)	C14—C19—H19B	109.5
C8—C9—C10	120.2 (3)	H19A—C19—H19B	109.5
C4—C9—C10	121.7 (2)	C14—C19—H19C	109.5
C9—C10—H10A	109.5	H19A—C19—H19C	109.5
C9—C10—H10B	109.5	H19B—C19—H19C	109.5
			
O11—C1—C2—C3	7.3 (3)	C2—C1—C12—C13	−2.3 (3)
C12—C1—C2—C3	−171.5 (2)	O11—C1—C12—C17	−1.5 (3)
C1—C2—C3—C4	178.5 (2)	C2—C1—C12—C17	177.34 (19)
C2—C3—C4—C5	−10.4 (3)	C17—C12—C13—C14	0.9 (3)
C2—C3—C4—C9	170.3 (2)	C1—C12—C13—C14	−179.4 (2)
C9—C4—C5—C6	−0.8 (4)	C12—C13—C14—C15	−0.6 (3)
C3—C4—C5—C6	179.9 (2)	C12—C13—C14—C19	178.3 (2)
C4—C5—C6—C7	0.5 (4)	C13—C14—C15—C16	−0.3 (4)
C5—C6—C7—C8	0.1 (4)	C19—C14—C15—C16	−179.2 (2)
C6—C7—C8—C9	−0.4 (5)	C14—C15—C16—C17	0.9 (4)
C7—C8—C9—C4	0.2 (4)	C15—C16—C17—O18	179.5 (2)
C7—C8—C9—C10	−179.0 (3)	C15—C16—C17—C12	−0.6 (4)
C5—C4—C9—C8	0.4 (3)	C13—C12—C17—O18	179.6 (2)
C3—C4—C9—C8	179.8 (2)	C1—C12—C17—O18	−0.1 (3)
C5—C4—C9—C10	179.6 (2)	C13—C12—C17—C16	−0.3 (3)
C3—C4—C9—C10	−1.0 (3)	C1—C12—C17—C16	−180.0 (2)
O11—C1—C12—C13	178.86 (19)		

### Hydrogen-bond geometry (Å, °)

**Table:**

*D*—H···*A*	*D*—H	H···*A*	*D*···*A*	*D*—H···*A*
O18—H18···O11	0.82	1.89	2.608 (3)	146
